# Combination of *Tripterygium wilfordii* Hook F and angiotensin receptor blocker synergistically reduces excretion of urinary podocytes in patients with type 2 diabetic kidney disease

**DOI:** 10.1080/13102818.2014.989727

**Published:** 2014-12-11

**Authors:** Ruixia Ma, Yan Xu, Wei Jiang, Wei Zhang

**Affiliations:** ^a^ Department of Nephrology, Affiliated Hospital of Medical College, Qingdao University, Qingdao, Shandong, P.R. China

**Keywords:** diabetic kidney disease, *Tripterygium wilfordii* Hook F, podocytes, transforming growth factor-β_1_

## Abstract

The aim of this study was to investigate whether *Tripterygium wilfordii* Hook F (TwHF) and irbesartan could synergistically affect the urinary excretion of podocytes and proteins in type 2 diabetic kidney disease (DKD) patients and the underlying mechanisms. Forty DKD patients were divided into a DI group (DKD patients treated with irbesartan alone) and a DTI group (DKD patients treated with Tripterygium wilfordii Hook F and irbesartan). Urinary podocytes were observed by immunofluorescence. Urinary levels of connective tissue growth factor (CTGF) and transforming growth factor-β_1_ (TGF-β_1_) were detected by enzyme-linked immunosorbent assay. Immunofluorescence indicated that shed podocytes were not detected in urine samples of normal controls, whereas the detection rate of urinary podocytes was 82.5% in DKD patients. Urinary CTGF and TGF-β_1_ levels were significantly higher in urinary podocyte-positive DKD patients than in urinary podocyte-negative patients. Furthermore, urinary podocyte excretion was closely correlated with urinary protein excretion and urinary CTGF/TGF-β_1_ levels. Treatments with TwHF and irbesartan significantly reduced the urinary excretion of proteins and podocytes, and decreased the urinary levels of CTGF and TGF-β_1_. Our results suggest that urinary podocyte excretion might serve as a predictor for DKD progression. TwHF/irbesartan combination could reduce the urinary excretion of proteins and podocytes synergistically in DKD patients, which might result from the synergistic inhibition of CTGF and TGF-β_1_ in urine.

## Introduction

Glomerular podocytes, a special type of epithelial cells, play an important role in maintaining the integrity of the filtration barrier in kidneys. Podocyte impairment has been shown to be involved in the development of proteinuria and the early pathological processes of diabetic kidney disease (DKD).[1,2] Recent studies indicate that the abnormal expression and distribution of podocyte surface marker proteins (such as nephrin and podocin), the reduction of podocytes, the loss of foot process fusion and other pathological changes may induce DKD and promote the disease progression.[3,4]

Since podocytes are terminally differentiated and cannot proliferate and transdifferentiate, the injury of these cells is not reversible. It is of great importance to investigate the impairment of podocytes in kidney disease. The detection of podocytes, however, requires renal tissue biopsy, which might be traumatic and could not be used for dynamic detection of podocyte lesions. Recent studies have found that podocytes shed in urine could serve as an indicator for disease progression in primary or secondary glomerular lesions, including DKD.[5,6]

Angiotensin receptor blocker (ARB) is one of the most widely used and effective drugs for type 2 DKD treatment. ARB exerts protective effects on renal podocytes and reduces urinary excretion of these cells.[7,8] On the other hand, our previous study [9] as well as reports from other laboratories [10,11] have also shown that triptolide, the effective component in immunosuppressant *Tripterygium wilfordii* Hook F (TwHF), could reduce proteinuria and protect the impaired podocytes in DKD model rats.[12,13] However, there are few reports on the effects of TwHF on urinary podocyte and protein excretion in DKD patients.[14]

In this study, we attempted to expand our studies on the effects of TwHF and irbesartan combination treatment on urinary excretion of podocytes and proteins in DKD patients, as well as on their synergistic protective effects on kidney and the underlying mechanisms of this protective effect. Our results might provide theoretical and experimental basis for the prevention and clinical treatment of DKD.

## Subjects and methods

### Patients

Forty patients with type 2 DKD were enrolled in this study, and 10 healthy volunteers were selected as normal controls. Criteria for DKD: patients suffering from type 2 diabetes (American Diabetes Association Criteria 1997), accompanied with proteinuria and diabetic retinopathy. The patients were clinically diagnosed to be suffering from DKD by ruling out the possibility of other kidney diseases. Proteinuria in 24 h > 0.5 g, serum creatinine <132 μmol/L, glycosylated haemoglobin (HbA1c) ≤7%, fasting blood glucose concentration <8 mmol/L, 2 h postprandial blood glucose concentration <12 mmol/L, sitting blood pressure in patients with hypertension ≤160/90 mmHg and sitting systolic blood pressure in patients with normal blood pressure ≥100 mmHg.

### Drug administration

The DKD patients were randomly divided into the following groups: an irbesartan (DI) group (*n* = 20) and a combination treatment (DTI) group (*n* = 20). After a washout period of six weeks, patients from the DI group received irbesartan treatment alone (150–300 mg/d) for 12 weeks, and patients from the DTI group were administered a combination of TwHF and irbesartan (1–2 mg/d TwHF + 150–300 mg/d irbesartan) for 12 weeks. During the drug administration period, antihypertensive drugs other than angiotensin-converting enzyme inhibitors (ACEI)/ARB (such as calcium antagonists, β-receptor antagonists and diuretics) were used to maintain the patient blood pressure below 140/90 mmHg. In order to exclude the interference of blood pressure and blood glucose, we adjusted the dosage levels of insulin to keep the patients’ fasting blood glucose concentration <8 mmol/L and 2 h postprandial blood glucose concentration <12 mmol/L.

### Collection of podocytes from urinary sediment

Fresh urine samples (100 mL) were collected from each patient 2–3 h after early morning bladder emptying. The samples were subjected to centrifugation at 3000 r/min for 10 min. Fifty microlitres urinary sediment was dropped on slides pre-coated with polylysine. After fixation in 4% paraformaldehyde for 15 min, the samples were air dried at room temperature.

### Indirect immunofluorescence

After washing three times (each time for 5 min) with 0.01 mol/L of phosphate buffered saline, the samples were incubated in 50 μL goat serum (1:10 dilution) at room temperature for 1 h. Then, mouse anti-human podocalyxin monoclonal antibody (1:50 dilution; Santa Cruz, CA, USA) was used for incubation. After washing, goat anti-mouse IgG (1:100 dilution; Boster, Wuhan, Hubei, China) was added and the samples were incubated at 35 °C for 30 min, followed by the incubation with streptavidin–biotin–avidin complex – fluorescein isothiocyanate (SABC-FITC; 1:100 dilution; Boster) at 35 °C for 30 min. The samples were then sealed with anti-quenching agents and observed with a fluorescence microscope (Leica DM 2500; Leica Microsystems, Wetzlar, Germany).

### Urinary podocyte counting

The podocytes were identified, observed and captured at a 200×–400× magnification. Twenty fields with high magnification from the left, right and centre parts of the slide were selected to conduct the podocyte counting.

### Determination of urinary protein levels

Urinary protein levels were determined with enzyme-linked immunosorbent assay (ELISA) kits (Santa Cruz, CA, USA), according to the manufacturer's instructions. Briefly, 50 μL of assay diluent was dispensed into each well, and 200 μL of standard or urine sample was added. After incubation at room temperature for 2 h, the wells were washed four times with washing buffer and mixed with 200 μL of cytokine conjugate. After a further incubation at room temperature for 2 h, the plate was washed with washing buffer. Then, 200 μL of substrate was added into each well for incubation at room temperature for 20 min, after which 50 μL of stop solution was added. Optical densities (450 nm) were recorded with a microplate reader (Tacan Safire 2, Tecan Trading AG, Männedorf, Switzerland).

### Statistical analysis

Data were expressed as mean ± standard deviation (SD). SPSS 16.0 software was used for statistical analysis. The *t*-test, paired *t*-test and χ^2^ test were used for the comparison between the two groups. Two-way analysis of variance (ANOVA) and Newman–Keuls *post hoc* test were used for multiple comparisons. Mann–Whitney *U* test was used for the non-parametric comparison. Correlation analysis was performed using Pearson correlation analysis. *P* < 0.05 was considered statistically significant.

## Results and discussion

### Urinary podocyte excretion detected in DKD patients

**Figure 1.**
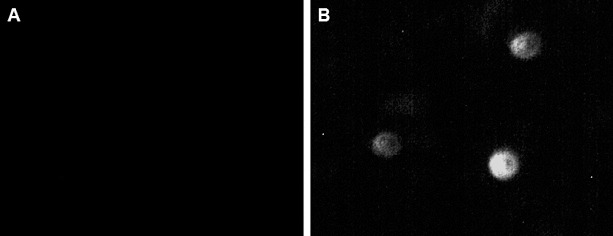
Immunofluorescence staining of podocytes in urine from normal controls (A) and DKD patients (B). Fluorescence indicated podocalyxin-positive cells (200 ×).

The basic information of the subjects was summarized in Table 1. To determine the urinary podocyte excretion in the DKD patients, we performed indirect immunofluorescence, using mouse anti-human podocalyxin monoclonal antibody (Figure 1). The staining indicated that shed podocytes were not detected in the urine of normal controls (Figure 1(A)), whereas they were found in urine samples from DKD patients, with a detection rate of 82.5% (Figure 1(B)) (*P* < 0.01). These results suggest that shed podocytes could be observed in urine from DKD patients, which might be used as an indicator for DKD.

**Table 1.** Comparison of physiological and biochemical indices of patients with diabetes.

Items	Control group	DI group	DTI group
*n*	10	20	20
Age	58.07 ± 5.33	57.40 ± 6.4	57.0 ± 5.1
gender (male/female)	5/5	10/10	11/9
BMI (kg/m^2^)	21.80 ± 1.9	23.9 ± 2.2	22.7 ± 1.4
DM history (years)	0	13.9 ± 2.1*	13.8 ± 3.0*
Proteinuria history (years)	0	2.9 ± 0.8*	3.0 ± 0.7*
FBG (mmol/L)	4.97 ± 0.33	6.4 ± 1.00*	6.9 ± 0.57*
HbA1c (%)	5.04 ± 0.4	7.6 ± 1.3*	7.5 ± 1.3*
MAP (mmHg)	93.41 ± 4.3	98.4 ± 3.0	96.4 ± 5.7
Scr (μmol/L)	96.53 ± 12.3	104.4 ± 14.6	106.5 ± 12.4
Ccr (min/ml)	84.17 ± 6.1	79.36 ± 4.5	78.1 ± 3.7
TG (mmol/L)	0.98 ± 0.4	2.4 ± 0.3*	2.4 ± 0.5*
CH (mmol/L)	4.82 ± 0.8	5.5 ± 0.2	5.4 ± 0.3
Proteinuria (g/d)	0.016 ± 0.05	2.6 ± 1.3*	2.7 ± 1.5*
WBC (×109/L)	6.26 ± 1.3	6.1 ± 1.1	5.8 ± 1.0
ALT (U/L)	36.67 ± 12.7	28.7 ± 12.4	29.8 ± 9.7
AST (U/L)	22.50 ± 5.5	20.8 ± 5.9	22.5 ± 7.3

Note: Compared with the control group, *P < 0.05. The DI group received irbesartan treatment alone and the DTI group received TwHF/irbesartan combination.

Abbreviations: BMI – body mass index; DM – diabetes mellitus; FBG – fasting blood glucose; HbA1c – glycosylated haemoglobin; MAP – mean aortic pressure; Scr – serum creatinine; Ccr – creatinine clearance; TG – triglyceride; CH – cholesterol; WBC – white blood cell; ALT – alanine aminotransferase; AST – aspartate aminotransferase.

### Increased urinary CTGF and TGF-β_1_ levels in DKD patients

**Figure 2.**
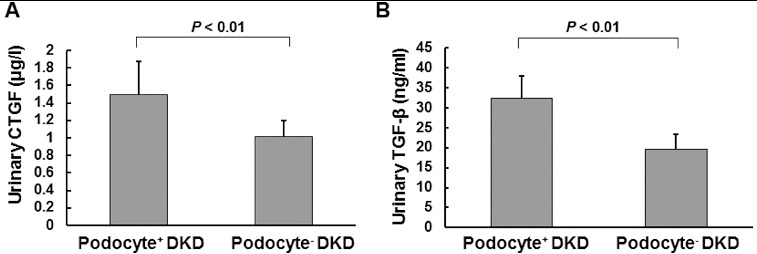
Urinary levels of CTGF (A) and TGF-β_1_ (B) in DKD patients, respectively, positive or negative for podocytes.

**Figure 3.**
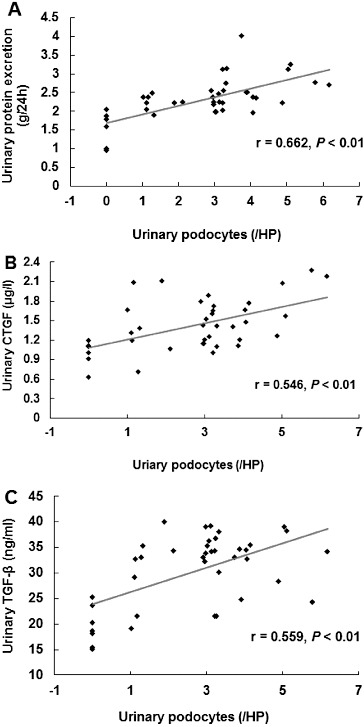
Correlation analysis between the urinary excretion of podocytes and proteins and the urinary levels of proteins (A), CTGF (B) and TGF-β_1_ (C) in DKD patients.

To investigate whether the urinary protein excretion was changed in DKD patients and to analyse its relationship to the urinary podocyte excretion, ELISA was performed. The urinary contents of connective tissue growth factor (CTGF) and transforming growth factor-β_1_ (TGF-β_1_) were detected. Our results indicated that the urinary levels of CTGF (Figure 2(A)) and TGF-β_1_ (Figure 2(B)) were significantly higher in DKD patients who were positive for urinary podocytes than in the podocyte-negative DKD patients (*P* < 0.01). Furthermore, correlation analysis showed that the urinary protein excretion was significantly correlated with urinary podocyte excretion (Figure 3(A)); (*r* = 0.662, *P* < 0.01). This result was consistent with our previous report.[13] Specifically, the urinary content of CTGF and TGF-β_1_ was significantly correlated (*P* < 0.01) with the urinary podocyte excretion, with correlation coefficients of 0.546 (Figure 3(B)) and 0.559 (Figure 3(C)), respectively. These results suggest that the urinary protein excretion is dramatically increased in podocyte-positive DKD patients.

### Reduced pathogenic changes in DKD patients after drug administration

**Figure 4.**
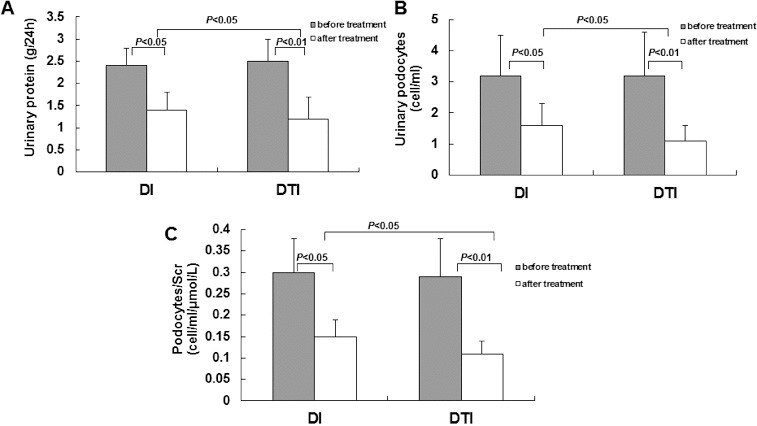
Effects of drug administration on pathogenic changes in DKD patients. Note: A 12 week period of irbesartan administration alone (DI) or combination treatment with TwHF/irbesartan (DTI). The urinary excretion of proteins (A) and podocytes (B) and the ratio of podocytes/serum creatinine (Scr) were detected before and after drug administration.

To investigate the effects of drug administration on pathogenic changes in DKD, these patients were subjected to administration of irbesartan alone (the DI group) or in combination with TwHF (the DTI group) as previously described.[13] Before drug administration, these two groups shared similar physiological and biochemical indices. There were no significant differences between these two groups in terms of age, gender, body mass index, history of diabetes, blood pressure, blood lipids, hepatic and renal function, blood routine and urinary protein excretion rate (Table 1) (*P* > 0.05). Our results showed that drug administration for 12 weeks significantly reduced the detection rates and amount of urinary protein and podocyte excretion (Figure 4(A) and 4(B)), and decreased the corrected ratio of urinary podocytes/serum creatinine (Figure 4(C)), compared with the data collected in the initial monitoring washout period. Moreover, TwHF/irbesartan combination treatment exhibited protective effects on urinary protein and podocyte excretion superior to those of irbesartan treatment alone (Figure 4(A)–(C)). In addition, 12 weeks treatment of TwHF exerted no significant side effects, such as liver and kidney dysfunction and bone marrow suppression (data not shown). These results suggested that drug administration could decrease the pathological changes in DKD, including the urinary excretion of podocytes and proteins, as well as the ratio of urinary podocytes/serum creatinine.

### TwHF/irbesartan combination exerts profound synergistic effects on reducing urinary protein levels in DKD patients

**Figure 5.**
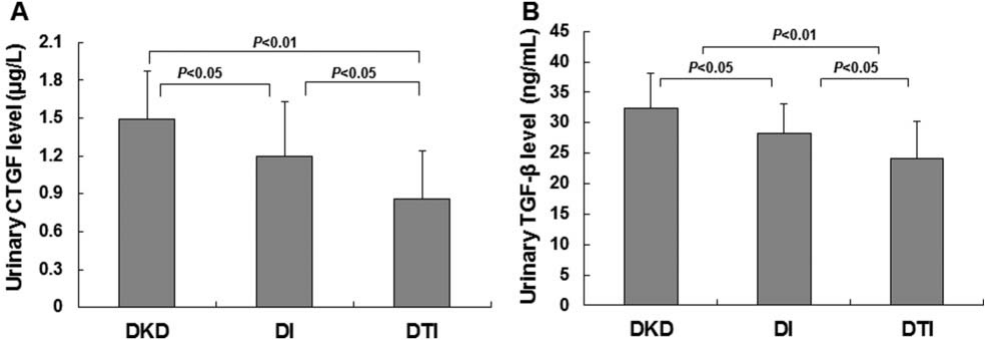
Synergistic effects of combined TwHF/irbesartan treatment on urinary levels of CTGF (A) and TGF-β1 (B); DKD patients before drug administration (DKD), DKD patients treated with irbesartan alone (DI); DKD patients treated with TwHF/irbesartan combination (DTI).

The above results suggest that the combined treatment of TwHF/irbesartan exhibited superior benefits. Next, we further checked the therapeutic effects of these drug treatments on urinary protein excretion in DKD patients. As expected, treatment either with irbesartan alone or with TwHF/irbesartan combination could significantly decrease the urinary CTGF level (Figure 5(A)): 1.20 ± 0.43 μg/L for the DI group (*P* < 0.05), 0.86 ± 0.38 μg/L for the DTI group (*P* < 0.01) vs. 1.49 ± 0.38 μg/L for the DKD group. A decrease in the urinary TGF-β_1_ level was also observed (Figure 5(B)): 28.28 ± 4.89 ng/mL for the DI group (*P* < 0.05), 24.14 ± 6.16 ng/mL for the DTI group (*P* < 0.01) vs. 32.45 ± 5.67 ng/mL for the DKD group. More importantly, the combination treatment led to significant reduction in the protein levels in urine samples, compared with the irbesartan treatment alone (Figure 5) (*P* < 0.05). These results suggest that combined treatment of TwHF and irbesartan exhibits synergistic effects on reducing urinary protein levels in DKD patients, which might be the underlying mechanism for the protective benefits of these drugs.

### Comparative analysis

DKD is one of the major chronic complications of diabetes and death causes. Thirty–forty per cent of end-stage renal disease is induced by DKD and the percentage continues to go up. DKD pathogenesis, however, has not yet been fully understood. Recent studies show that DKD pathogenesis is closely linked with various pathways and/or multi-targets, including abnormal glucose and lipid metabolism, chronic inflammation, oxidative stress, immune system activation and podocyte impairment.[15,16] Furthermore, the impairment in podocyte structure and function might be the central link in DKD pathogenesis.[1–4] Podocytes are terminally differentiated cells with limited proliferative potential.[17] Impaired podocytes could lead to glomerular sclerosis. Therefore, the prevention of podocyte-related pathogenesis has become a hot topic for research on DKD treatment.

Angiotensin-converting enzyme inhibitor (ACEI) and angiotensin receptor antagonist (ARB) are the most widely accepted drugs for DKD treatment. However, the efficacy of these drugs is not significant in DKD patients with proteinuria. Recently, reports from our and other laboratories have shown that anti-inflammatory, antioxidant immunosuppressants could exert beneficial effects for DKD,[12,18,19] which has attracted considerable attention. In addition to the treatment for metabolic disorders and haemodynamic abnormalities, the combination of anti-inflammatory, antioxidant immunosuppressants might provide a novel effective therapeutic strategy for DKD.

TwHF is the only plant medicine with immunosuppressive potential, which could exert anti-inflammatory, anti-proliferative, antioxidant and immunosuppressive effects to reduce urinary protein excretion in a variety of primary and secondary glomerular diseases. Recently, *in vitro* and *in vivo* studies have found that triptolide, the active ingredient in TwHF, could protect against DKD via various pathways and with multi-targets, including the regulation of metabolic abnormalities, anti-inflammation, antioxidant action and the protection of podocytes.[11] Our previous study has shown that triptolide could significantly reduce urinary protein excretion, improve podocyte ultrastructure, decrease renal podocyte proteins of nephrin and podocin and reduce the levels of pro-inflammatory factors of CTGF and TGF-β_1_ in DKD model rats. The triptolide/irbesartan combination could exhibit a synergistic effect in reducing urinary excretion of proteins and podocytes.[11,13] Moreover, Song et al. [10] found that TwHF could reduce urinary excretion of monocyte chemotactic protein, suppress inflammatory response, decrease proteinuria and improve renal function in DKD patients. However, whether the combination of TwHF and irbesartan could exhibit a synergistic effect on the urinary excretion of proteins and podocytes in DKD patients has not been reported.

In this paper, we investigated the effects of TwHF/irbesartan combination on the urinary excretion of proteins and podocytes in DKD patients. Our results indicated that the combination of TwHF and irbesartan could dramatically reduce the urinary excretion of proteins and podocytes in DKD patients, with a significantly stronger effect compared with irbesartan treatment alone. In order to determine the possible mechanisms through which TwHF/irbesartan combination reduced urinary excretion of proteins and podocytes in DKD patients, we investigated the contents of CTGF and TGF-β_1_ in urine samples. Results showed that the CTGF and TGF-β_1_ levels in urine were significantly higher in DKD patients positive for urinary podocytes than podocyte-negative DKD patients. Moreover, the amount of urinary podocytes was correlated with urinary CTGF and TGF-β_1_ levels, indicating that there was a close relationship between urinary CTGF/TGF-β_1_ levels and podocyte impairment in DKD. These findings are in consistence with previous studies in which podocyte impairment in DKD has been linked to CTGF and TGF-β_1_ over-expression in renal tissues.[20,21] Takano et al. [22] found that activated macrophages could produce pro-inflammatory cytokines of interleukin-1 and tumor necrosis factor-α and down-regulate the expression of nephrin via the phosphatidylinositol-3-kinase/protein kinase B pathway *in vitro*. CTGF also plays a key role in DKD pathogenesis.[22] CTGF has been shown to be involved in the intra-cellular and extra-cellular TGF-β_1_ signalling cascade through various mechanisms. CTGF serves as a downstream effector in the TGF-β_1_ signalling pathway, promoting the expression of growth factors. CTGF also inhibits the transcription of Smad7 through tyrosine receptor kinase to increase TGF-β_1_ signalling. Moreover, CTGF has been found to indirectly accumulate the cell proliferation and extracellular matrix synthesis-stimulating effects of TGF-β_1_, and to deteriorate the podocyte impairment caused by TGF-β_1_, in combination with the bone morphogenetic protein (BMP)-7 signalling pathway. Based on the mechanisms of irbesartan and TwHF, the drug combination could suppress the expression levels of CTGF and TGF-β_1_ in renal tissues synergistically, providing an effective way to treat DKD. We investigated the effects of TwHF and irbesartan on urinary excretion of CTGF and TGF-β_1_ in DKD patients, especially with the concern of urinary podocyte excretion. Our results showed that drug treatment decreased the urinary levels of CTGF and TGF-β_1_, with the TwHF/irbesartan combination exhibiting stronger effects, suggesting that the urinary protective effect of TwHF may be related to the inhibition of CTGF and TGF-β_1_ expression in renal tissue.

In this study, we also investigated the side effects of TwHF at the dosage of 1–2 mg/kg/d. Our results showed that 1–2 mg/kg/d TwHF administration for 12 weeks has no significant effects on leukocytes, liver enzymes and renal function, suggesting that TwHF at this dosage does not exert side effects, such as bone marrow suppression and liver and/or kidney damage. This finding is in line with the results of Li and Liu [23], who report that the immunosuppressive effect of TwHF is different from all the other immunosuppressants. *Tripterygium* glycosides provide a strong immunosuppressive effect, without causing serious damages to normal immune system, inducing tumours, or inducing infections. *Tripterygium* glycosides strongly inhibited T-cells, with a weak action on resting cells. Therefore, a short period of *Tripterygium* glycosides administration to DKD patients would not produce serious adverse reactions.

Taken together, our results indicate that the measurement of urinary podocytes and cytokines may provide a simple, non-invasive method to monitor DKD pathogenesis, determine the responses to drug treatment and estimate the prognosis. Our study provides the basis for possible future integration of the combination of TwHF and ARB in clinical treatment of DKD. Of course, further studies with expanded sample size and elongated observation period are still needed.

## Conclusions

Our results provided evidence that the measurement of urinary podocytes and cytokines may provide a simple, non-invasive method to monitor DKD pathogenesis, determine the responses to drug treatment and conduct the prognosis estimation. TwHF/irbesartan combination could reduce the urinary excretion of proteins and podocytes. The possible mechanisms may be related to the reduction of CTGF and TGF-β_1_ contents. The drug combination exerts a synergistic effect on renal protection, without significant adverse reactions at the dosage level. Our study provides the basis of the combination of TwHF and ARB in clinical treatment of DKD. Of course, further studies with expanded sample size and elongated observation period are still needed.
